# Genomic characterization of clinical and environmental *Vibrio cholerae* O1 and O139 isolates in Jiaxing, China, with identification of a *ctx*-positive O139 strain harboring an IncC plasmid

**DOI:** 10.3389/fmicb.2026.1751786

**Published:** 2026-04-15

**Authors:** Miaomiao Jia, Ping Li, Yong Yan, Qiong Wu, Xuejuan Liu, Lei Gao, Guoying Zhu, Zhongwen Chen

**Affiliations:** 1 Jiaxing Key Laboratory of Pathogenic Microbiology, Jiaxing Center for Disease Control and Prevention, Jiaxing, China; 2 Department of Clinical Laboratory, The Second Affiliated Hospital of Xiamen Medical College, Xiamen, Fujian, China

**Keywords:** antimicrobial susceptibility, genetic relatedness, IncC plasmid, O139 serogroup, *Vibrio cholerae*, virulence factors

## Abstract

**Introduction:**

*Vibrio cholerae* serogroups O1 and O139 are responsible for epidemic and pandemic cholera. Although the pathogenic potential and genomic diversity of *V. cholerae* strains have been extensively studied in endemic regions, limited genomic data are available for more developed regions such as Jiaxing.

**Methods:**

In this study, 15 *V. cholerae* O1 and O139 isolates (eight clinical and seven environmental) collected between 2021 and 2024 were analyzed. Antimicrobial susceptibility testing (AST) was performed, and whole-genome sequencing was conducted. Comparative genomic analyses were used to characterize antimicrobial resistance (AMR) determinants, virulence-associated genes, and population structure. Core genome multilocus sequence typing (cgMLST) was applied to assess genetic relatedness.

**Results:**

All strains were susceptible to ciprofloxacin, trimethoprim–sulfamethoxazole, tigecycline, and amikacin. Elevated MIC values were observed for colistin; however, no interpretive criteria are available for *V. cholerae*. By contrast, high resistance rates were observed for streptomycin, chloramphenicol, and azithromycin. Resistance genes, including *qnr*, *tet*, *mph*, and *sul*, were widely distributed, while *bla* genes were absent. One clinical O139 strain, VC0827, was found to harbor the *ctxAB* genes, a truncated CTX prophage, and an IncC plasmid (pVC0827), which carried several antimicrobial resistance genes including *tet*(*A/B/M*), *flor*, *sul2*, and *msr*(*E*). cgMLST analysis revealed three main clusters; VC0827 clustered closely with seventh-pandemic reference strains and shared sequence type ST69. The observed gene duplications (e.g., *ace* and *zot*) in VC0827 may enhance its toxigenic potential.

**Conclusion:**

This study highlights the genomic diversity and resistance profiles of *V. cholerae* in Jiaxing. The identification of a potentially virulent, multidrug-resistant O139 strain underscores the need for continuous genomic surveillance to monitor the emergence of toxigenic lineages and horizontal gene transfer.

## 1 Introduction

*Vibrio cholerae* is a Gram-negative, curved rod-shaped bacterium that causes cholera (a severe diarrheal illness) and is transmitted through the consumption of contaminated food or water (Monir et al., 2023). This bacterium has been divided into more than 200 serogroups based on the lipopolysaccharide component (O antigen) in the cell wall (Kaper et al., 1995). Among them, only serogroups O1 and O139 are associated with epidemic and pandemic cholera outbreaks (Camacho et al., 2018; Mahapatra et al., 2014). The World Health Organization estimates that cholera affects between 1.3 and 4 million people worldwide each year, resulting in 21,000–143,000 deaths (Yuan et al., 2022).

To date, seven cholera pandemics have occurred, all linked to the O1 serogroup, the first of which was in 1817 (Weil and Ryan, 2018). The O1 group is further divided into two biotypes: classical and El Tor (Kanungo et al., 2022). In the early 1990s, a distinctive non-O1 strain of *V. cholerae* was discovered in India, causing a disease similar to that caused by the O1 El Tor strain (Kanungo et al., 2022). This strain did not react with any of the 138 somatic antisera known at the time and was thus categorized into a new serogroup, later designated O139 (Ramamurthy et al., 2022). However, researchers subsequently discovered that the epidemic O139 clone originated from a seventh pandemic O1 strain, in which the O1 antigen gene cluster had been replaced by the O139 O antigen gene cluster (Ramamurthy et al., 1993). Therefore, the O139 strain is genetically part of the seventh pandemic lineage and shares the same sequence type (Mukhopadhyay et al., 1998).

In recent years, the rise in antimicrobial resistance among *V. cholerae* strains has become a global concern (Kitaoka et al., 2011). Resistance to commonly used antibiotics such as tetracyclines, fluoroquinolones, and macrolides has been increasingly reported, compromising treatment effectiveness and complicating outbreak control (Verma et al., 2019). Whole-genome sequencing has facilitated the identification of AMR genes, which are often located on mobile genetic elements, including integrative and conjugative elements and plasmids (Bag et al., 2019; Carraro et al., 2016; Lassalle et al., 2023). Among these, IncC plasmids have gained attention because of their ability to carry and disseminate multiple resistance genes across bacterial species (Kitaoka et al., 2011; Verma et al., 2019; Wang et al., 2018). Some studies have revealed the presence of multiple *ctxAB* copies, variations in *tcpA*, and the acquisition of novel virulence islands, which may enhance the pathogenic potential of specific strains (Bhandari et al., 2023).

Despite extensive studies in endemic regions, limited genomic data are available for clinical and environmental *V. cholerae* O1 and O139 strains in certain areas of China, including Jiaxing. In this study, we performed whole-genome sequencing of 14 *V. cholerae* O1 isolates and one *V. cholerae* O139 isolate collected in Jiaxing, Zhejiang Province, China, between 2021 and 2024. We investigated their AMR gene profiles, virulence factors, and the presence of IncC plasmids. Notably, one O139 strain was identified as *ctxAB*-positive and carried an IncC plasmid. Furthermore, comparative genomic and core genome multilocus sequence typing (cgMLST) analyses were conducted to characterize the genetic features of these isolates and to explore their genetic relatedness with *V. cholerae* strains from other regions globally.

## 2 Methods

### 2.1 Bacterial strains and genomic characterization

A total of 15 *V. cholerae* isolates collected between 2021 and 2024 in Jiaxing, Zhejiang Province, China, were included in this study. Among these isolates, eight were obtained from clinical cases of diarrhea, and seven were recovered from environmental sources, including river water and pond water. All isolates were identified as *V. cholerae* using standard microbiological methods and were stored at −80 °C until further analysis. Whole-genome sequencing was performed on the 15 isolates collected in this study. In addition, six publicly available genome sequences of *V. cholerae* were retrieved from the NCBI database for comparative genomic analysis. These reference genomes were not sequenced in this study but were included to provide evolutionary context. More details on the isolates are provided in Table 1 (Jia et al., 2025). All patient data were treated as confidential. The isolates were collected as part of routine surveillance activities, and detailed clinical treatment information for the patients was not available; therefore, correlations between antimicrobial susceptibility results and treatment regimens could not be evaluated.

**Table 1:** Complete genomes of *V. cholerae* O1 and O139 used in this study.

Strain	Serogroup	Year	Location	Source	Sample type	Genome sequence reference
C5	O1	1957	Indonesia	GCA_001887395	–	Luo et al. (2024) and Suarez et al. (2008)
E9120	O1	1961	Indonesia	GCA_001887655	–	Luo et al. (2024) and Suarez et al. (2008)
E1162	O1	1962	China	GCA_001887495	–	Luo et al. (2024) and Suarez et al. (2008)
CRC711	O1	1964	India	GCA_001887435	–	Luo et al. (2024) and Suarez et al. (2008)
M806	O1	1964	India	GCA_001887455.1	–	Luo et al. (2024) and Suarez et al. (2008)
E7946	O1	1978	Bahrain	GCA_013085165	–	Luo et al. (2024) and Suarez et al. (2008)
VC0451	O1	2021	China	Clinical	Stool	This study
VC0452	O1	2021	China	Clinical	Stool	This study
VC0453	O1	2021	China	Clinical	Stool	This study
VC0298	O1	2022	China	Clinical	Rectal swab	This study
VC0360	O1	2023	China	Clinical	Stool	This study
VC0861	O1	2024	China	Clinical	Rectal swab	This study
VC0898	O1	2024	China	Environmental	Pond water	This study
VC0899	O1	2024	China	Environmental	Pond water	This study
VC0901	O1	2024	China	Environmental	River water	This study
VC0902	O1	2024	China	Environmental	River water	This study
VC0904	O1	2024	China	Environmental	River water	This study
VC0968	O1	2024	China	Environmental	Pond water	This study
VC0969	O1	2024	China	Environmental	Pond water	This study
VC0827	O139	2024	China	Clinical	Stool	This study
VC1026	O1	2024	China	Clinical	Rectal swab	This study

### 2.2 Antimicrobial susceptibility testing

The minimum inhibitory concentrations (MICs) of chloramphenicol, co-trimoxazole (trimethoprim–sulfamethoxazole), colistin, ertapenem, meropenem, cefotaxime, ceftazidime, ceftazidime–avibactam, tetracycline, tigecycline, ciprofloxacin, nalidixic acid, azithromycin, streptomycin, ampicillin, and ampicillin–sulbactam were determined using the broth micro-dilution method. Antimicrobial susceptibility testing was performed using a Sensititre custom broth microdilution plate (Thermo Fisher Scientific, Waltham, MA, United States) according to the manufacturer’s instructions. Bacterial suspensions were prepared to a turbidity equivalent to 0.5 McFarland standard and inoculated into cation-adjusted Mueller–Hinton broth. Plates were incubated at 35 °C ± 2 °C for 16–20 h under aerobic conditions. MIC values were interpreted in accordance with the Clinical and Laboratory Standards Institute (CLSI) guidelines (CLSI M100, 2023 edition; M45, 2016 edition). As no CLSI interpretive criteria are defined for colistin against *V. cholerae*, colistin MICs were not interpreted as susceptible or resistant. Colistin MIC values were not included in the classification of Multidrug-resistant (MDR) phenotypes. *Escherichia coli* ATCC 25922, *Enterococcus faecalis* ATCC29212, *Pseudomonas aeruginosa* ATCC27853, and *Staphylococcus aureus* ATCC29213 were used as quality control strains for AST. MDR was defined as resistance to at least three antimicrobial classes.

### 2.3 Whole genome sequencing

Total genomic DNA was extracted from overnight (16–18 h) cultures of all strains using the QIAamp DNA Mini Kit (Qiagen, Hilden, Germany) following the manufacturer’s instructions. DNA quality and concentration were assessed using a NanoDrop spectrophotometer and agarose gel electrophoresis. Whole-genome sequencing was performed on the NextSeq 550 platform (Illumina, San Diego, CA, United States) using paired-end reads (2 × 150 bp). Raw sequencing reads were subjected to quality control using FastQC (v0.11.9), and low-quality reads and adapter sequences were removed using Trimmomatic (v0.39) with default parameters. High-quality reads were then assembled *de novo* using SPAdes (v3.15.5) with default settings. No additional post-assembly polishing or cleaning steps were performed beyond standard quality assessment. Assembly quality was evaluated based on standard metrics, including N50 and total assembly length (Jia et al., 2025).

### 2.4 Detection of antimicrobial resistance and virulence genes

Antimicrobial resistance genes were identified using ResFinder (version 4.1) available at the Center for Genomic Epidemiology^1^ (Bhandari et al., 2023). Virulence genes were detected using CholeraFinder through the CGE platform. Default parameters were applied, with a minimum threshold of 95% sequence identity and 60% coverage for gene detection, to ensure reliable identification of functionally relevant genes (Kaper et al., 1995).

### 2.5 Core genome multilocus sequence typing analysis

To investigate the genetic relatedness among the isolates, core genome multilocus sequence typing (cgMLST) analysis was performed. This approach is based on allelic differences and is used for clustering rather than phylogenetic inference. The analysis included the 15 *V. cholerae* isolates sequenced in this study along with six reference genome sequences retrieved from the NCBI database (Luo et al., 2024). The assembled genome sequences were uploaded to the BacWGSTdb platform.^2^ Allelic profiles were determined based on the cgMLST scheme implemented in the database, and the genetic relationships among the isolates were inferred using a minimum spanning tree constructed according to allelic differences. The resulting tree was visualized and annotated using the Interactive Tree of Life (iTOL) version 4^3^ (Bhandari et al., 2023).

### 2.6 Analysis and comparison of *ctx* phages and plasmids

The sequence was submitted to the PHASTER online web interface^4^ using default settings to detect the presence of *V. cholerae* phages (Bhandari et al., 2023). Plasmids were identified using the PlasmidFinder tool^5^ (Jia et al., 2025). Gene organization was visualized using Easyfig v2.2.5 (local version), based on BLASTN comparisons of assembled genomic contigs and reference sequences. Sequence similarity analysis of CTX prophage regions was performed using BLASTN against the NCBI nucleotide database^6^ with default parameters.

## 3 Results

### 3.1 Antimicrobial resistance gene profiles and genotypic profiles

High susceptibility rates were observed for several antibiotics, including ciprofloxacin, trimethoprim–sulfamethoxazole, tigecycline, and amikacin, to which all isolates were susceptible (100%) (Table 2). Similarly, most isolates were susceptible to cephalosporins and carbapenems, including cefotaxime, ceftazidime–avibactam, ertapenem, and meropenem. By contrast, high resistance rates were observed for several other antibiotics. All isolates exhibited high MIC values for colistin; however, no interpretive criteria are available for *V. cholerae*. A high proportion of isolates were resistant to streptomycin (73.3%) and chloramphenicol (60.0%). Moderate resistance was observed for azithromycin (53.3%) and tetracycline (40.0%). Nalidixic acid resistance was detected in 33.3% of the isolates. Notably, differences in resistance patterns were observed between clinical and environmental isolates. Environmental isolates showed higher resistance rates to several antibiotics, including chloramphenicol, streptomycin, and azithromycin, whereas clinical isolates generally displayed higher susceptibility rates.

**Table 2:** Antimicrobial resistance profile of 15 isolated strains from 2021 to 2024.

		Antibiotic resistance profile								
		Total (*n* = 15)			Clinical (*n* = 8)			Environment (*n* = 7)		
Antimicrobial category		S	I	R	S	I	R	S	I	R
Beta-lactams	AMP	13 (86.7%)	0	2 (13.3%)	6 (75.0%)	0	2 (25.0%)	7 (100%)	0	0
	AMS	14 (93.3%)	0	1 (6.7%)	7 (87.5%)	0	1 (12.5%)	7 (100%)	0	0
	CZA	15 (100%)	0	0	8 (100%)	0	0	7 (100%)	0	0
	CTX	15 (100%)	0	0	8 (100%)	0	0	7 (100%)	0	0
	CTZ	14 (93.3%)	0	1 (6.7%)	7 (87.5%)	0	1 (12.5%)	7 (100%)	0	0
Carbapenems	ETP	14 (93.3%)	0	1 (6.7%)	7 (87.5%)	0	1 (12.5%)	7 (100%)	0	0
	MEM	14 (93.3%)	0	1 (6.7%)	7 (87.5%)	0	1 (12.5%)	7 (100%)	0	0
Polypeptide	CT	NA	NA	NA	NA	NA	NA	NA	NA	NA
Aminoglycoside	AMI	15 (100%)	0	0	8 (100%)	0	0	7 (100%)	0	0
	STR	3 (20.0%)	1 (6.7%)	11 (73.3%)	3 (37.5%)	1 (12.5%)	4 (50.0%)	0	0	7 (100%)
Macrolide	AZM	7 (46.7%)	0	8 (53.3%)	6 (75.0%)	0	2 (25.0%)	1 (14.3%)	0	6 (85.7%)
Tetracycline	TET	6 (40.0%)	3 (20.0%)	6 (40.0%)	6 (75.0%)	0	2 (25.0%)	0	3 (42.9%)	4 (57.1%)
Quinolone	CIP	15 (100%)	0	0	8 (100%)	0	0	7 (100%)	0	0
Antifolate	SXT	15 (100%)	0	0	8 (100%)	0	0	7 (100%)	0	0
	NAL	10 (66.7%)	0	5 (33.3%)	6 (75.0%)	0	2 (25.0%)	4 (57.1%)	0	3 (42.9%)
Phenicol	CHL	6 (40.0%)	0	9 (60.0%)	6 (75.0%)	0	2 (25.0%)	0	0	7 (100%)
Glycylcyclins	TIG	15 (100%)	0	0	8 (100%)	0	0	7 (100%)	0	0

CHL, Chloramphenicol; SXT, Cotrimoxazole; CT, Colistin; ETP, Ertapenem; MEM, Meropenem; CTX, Cefotaxime; CTZ, Ceftazidime; CZA, Ceftazidime-Avibactam; TET, Tetracycline; TIG, Tigecycline; CIP, Ciprofloxacin; NAL, Nalidixic acid; AMI, Amikacin; AZM, Azithromycin; STR, Streptomycin; AMP, Ampicillin; AMS, Ampicillin-sulbactam.

Colistin MIC values were determined but not interpreted according to CLSI criteria.

**Figure 1:**
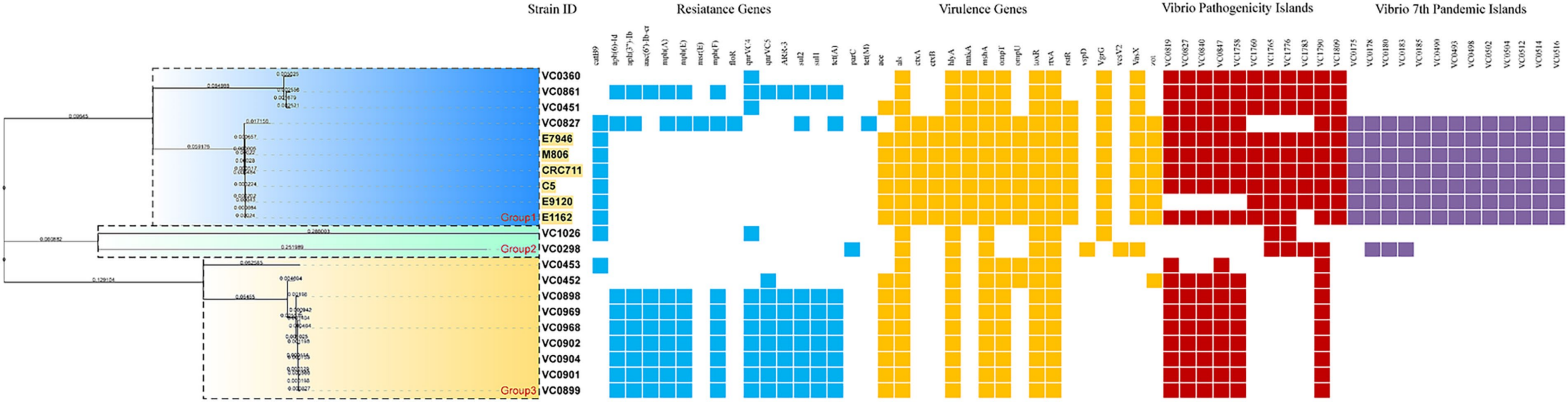
Minimum spanning tree based on cgMLST allelic profiles showing the genetic relationships among 21 *Vibrio cholerae* strains. Antimicrobial resistance genes, virulence genes, *Vibrio* pathogenicity islands (VPIs), and seventh pandemic islands (VSPs) are indicated on the right. Strains highlighted in yellow represent reference genomes retrieved from the NCBI database. Numbers on the connecting lines (nodes) represent the number of allelic differences between isolates based on the cgMLST scheme, reflecting their genetic relatedness.

Genotypic analysis revealed that several antimicrobial resistance genes were widely distributed among the isolates (Figure 1; Table 3). Quinolone resistance genes (*qnr*) and tetracycline resistance genes (*tet*) were detected in 66.7% of the strains, while macrolide resistance genes (*mph*) were present in 60.0% of the isolates. The chloramphenicol resistance gene *catB9* was detected in three isolates (3/15, 20.0%). Interestingly, although resistance to certain β-lactam antibiotics was observed phenotypically, no β-lactamase genes (*bla*) were detected in these genomes. In addition, 9 isolates carried genes associated with resistance to chloramphenicol (*floR*), sulfamethoxazole (*sul*), and streptomycin (*strA* and *strB*). According to the standard definition of MDR, 11 of the 15 isolates were classified as MDR strains. One clinical isolate (VC0827) was found to harbor the integrative mobile element *intSXT*.

**Table 3:** Distribution of antimicrobial resistance genes and virulence genes among 15 *V. cholerae* isolates collected from 2021 to 2024.

Gene	Presence (%)
Antimicrobial resistance genes (%)	
*catB9*	20.0%
*strB*	60.0%
*strA*	60.0%
*aac (6′)-Ib-cr*	53.3%
*mph(A)*	60.0%
*mph(E)*	60.0%
*msr(E)*	6.0%
*mph(F)*	60.0%
*floR*	6.0%
*qnrVC4*	73.3%
*qnrVC5*	60.0%
*ARR-3*	53.3%
*sul2*	60.0%
*sul1*	53.3%
*tet(A)*	60.0%
*parC*	6.0%
*tet(M)*	6.0%
Virulence genes (%)	
*ace*	60.0%
*als*	60.0%
*ctxA*	6.0%
*ctxB*	6.0%
*hlyA*	100%
*makA*	26.6%
*mshA*	100%
*ompT*	86.6%
*ompU*	20.0%
*toxR*	100%
*rtxA*	100%
*rstR*	13.3%
*vspD*	6.0%
*Vg*	33.3%
*vcsV2*	6.0%
*VasX*	33.3%
*zot*	13.3%

Percentages indicate the proportion of isolates carrying each gene.

### 3.2 Virulence gene profiles

As illustrated in Figure 1 and summarized in Table 3, the sequenced *V. cholerae* strains exhibited diverse virulence gene profiles. All 15 O1 and O139 isolates from Jiaxing carried several common virulence-associated genes, including *als*, *hlyA*, *mshA*, *toxR*, and *rtxA*. Notably, none of the strains harbored the heat-stable enterotoxin gene (*stn*). Only one O139 isolate (VC0827) carried the *ctxAB* genes and multiple virulence genes, including *hlyA*, *zot*, *vgrG, vasX*, *rtxA*, *rtxR*, *ompU*, *ompT*, *toxR*, *mshA*, *makA*, and *als*. This strain contained a truncated CTX prophage region, as well as *zot*, *ace*, and a VPI region. Moreover, VC0827 possessed the *VC2346* gene, a genetic marker associated with seventh-pandemic lineages. Additionally, none of the isolates carried type III secretion system genes (*vcsV2* and *vcsC2*), whereas six strains (VC0298, VC0360, VC0451, VC0827, VC0861 and VC1026) were positive for type VI secretion system–related genes (*vasX* and/or *vgrG*).

### 3.3 CTX prophage analysis of O139 *Vibrio cholerae* strain VC0827

**Figure 2:**
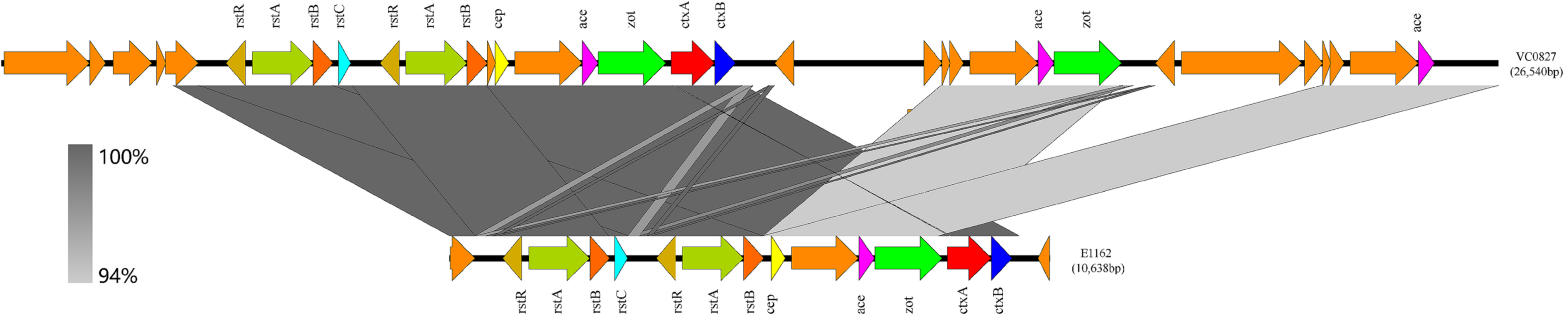
Comparative analysis of CTX prophage regions. The CTX prophage sequence from the O139 strain VC0827 identified in this study was compared with the closely related CTX prophage from the O1 strain E1162 previously isolated in China.

A single CTX prophage was identified in one clinical strain, VC0827 (serogroup O139). As shown in Figure 2, the genomic architecture of the CTX prophage in VC0827 was similar to that of the seventh-pandemic reference strain E1162, isolated in Shanghai, China. The CTX prophage in VC0827 was 26,540 bp in length, with a G + C content of 47.69%. Alignment with the reference sequence (E1162) showed a query coverage of 64%. Notably, VC0827 carried three copies of the *ace* gene and two copies of the *zot* gene, whereas E1162 contained only one copy of each. The orientation and gene arrangement of these loci in VC0827 were consistent with those in the reference strain.

### 3.4 Basic characteristics of pVC0827

**Figure 3:**
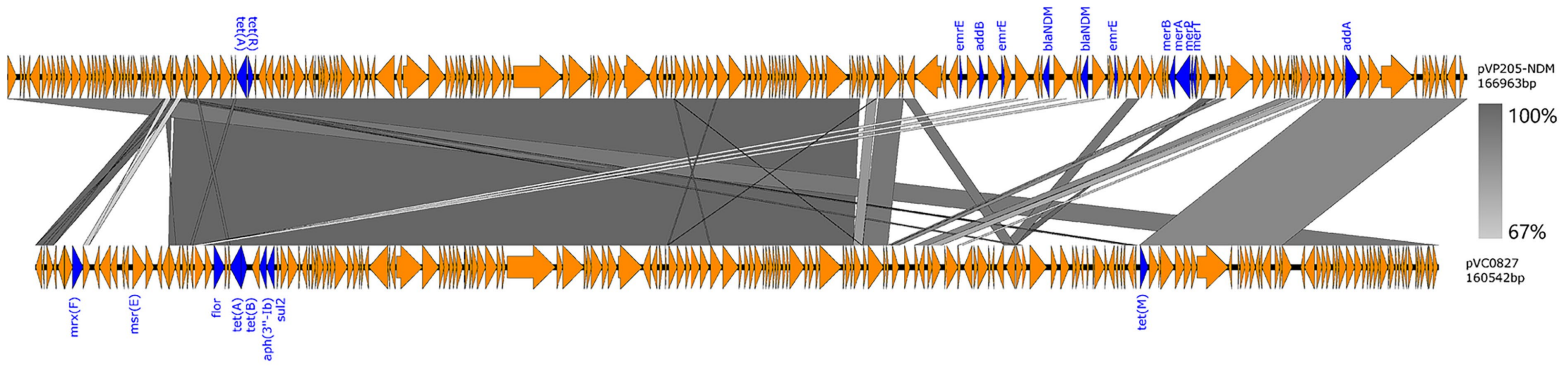
Comparison of the IncC plasmid pVC0827 identified in this study with the closely related plasmid pVP205-NDM from *Vibrio parahaemolyticus* strain VP205. Antibiotic resistance genes are shown in blue. Arrows indicate the position and directions of transcription genes. Regions with >99% sequence homology are indicated in gray. Triangles indicate truncated genes.

One isolate, VC0827, harbored an IncC plasmid. This plasmid showed partial sequence similarity to a reference plasmid from *Vibrio parahaemolyticus* strain VP205 isolated in China in 2024 (pVP205-NDM, 166,963 bp, 199 predicted coding sequences), with a query coverage of 51% and a nucleotide identity of 99.25% (Figure 3). The reference plasmid carries *tet(A), tet(R)*, *addB*, *merB*, *merA*, *merP*, *merT*, *addA*, two copies of *bla*_NDM_, and three copies of *emrE*. The plasmid from VC0827, designated pVC0827, was assembled as a contig of 160,542 bp with a G + C content of 51.5%, slightly lower than that of the reference (52.8%). A total of 197 coding sequences were predicted, and pVC0827 carried multiple antimicrobial resistance genes, including *mrx(F)*, *msr(E)*, *aph(3″)-Ib*, *sul2*, *flor*, *tet(A), tet(B)*, and *tet(M)*.

### 3.5 cgMLST-based clustering analysis

A minimum spanning tree based on cgMLST allelic profiles was constructed to investigate the genetic relatedness among the isolates. The analysis included 15 *V. cholerae* isolates from this study together with six reference genomes retrieved from the NCBI database (Table 1). As shown in Figure 1, the strains were grouped into three main clusters (Groups 1, 2, and 3). Allelic differences between closely related isolates within clusters were generally low, whereas substantially larger allelic distances were observed between clusters, supporting their genetic separation. The isolates were unevenly distributed among the three groups. Group 1 was further divided into two clusters. One cluster included three clinical isolates collected in 2021, 2023, and 2024 (VC0360, VC0861, and VC0451, respectively). The other cluster comprised the *ctx*-positive isolate VC0827 (2024) together with six reference strains, all belonging to sequence type ST69. Group 2 consisted of two clinical isolates collected in 2023 (VC0298) and 2024 (VC1026). Group 3 included two clinical isolates from 2021 (VC0453 and VC0452) and several environmental isolates from 2024 (VC0898, VC0899, VC0901, VC0902, VC0904, VC0968, and VC0969).

## 4 Discussion

*V. cholerae* is a Gram-negative bacterium commonly found in aquatic environments worldwide (Kanungo et al., 2022). The O1 and O139 serogroups are the primary causes of cholera and represent major waterborne pathogens associated with diarrheal diseases (Luo et al., 2022; Hounmanou et al., 2023). However, the evolutionary dynamics and pathogenic mechanisms of O1 and O139 strains remain less well characterized in certain regions, including China. Our previous study on clinical and environmental *V. cholerae* non-O1/non-O139 strains isolated from Jiaxing, China, revealed the presence of potentially pathogenic environmental strains capable of causing human illness (Jia et al., 2025). In this study, we performed genomic and phenotypic characterization of 15 O1 and O139 *V. cholerae* isolates from clinical and environmental sources collected in Jiaxing, China, between 2021 and 2024. Through antimicrobial susceptibility testing, whole-genome sequencing, and comparative genomic analyses, we provide insights into the resistance profiles, virulence potential, and genetic relatedness of these strains.

Early studies have reported that O1/O139 *V. cholerae* strains exhibit relatively high resistance rates (up to 50%) to trimethoprim–sulfamethoxazole and ciprofloxacin (Yuan et al., 2022; Yu et al., 2012; Das et al., 2020). Between 2000 and 2020, resistance trends were observed for nalidixic acid, trimethoprim–sulfamethoxazole, furazolidone, and tetracycline increased, whereas resistance to several other antibiotics, including ampicillin and chloramphenicol, decreased (Yuan et al., 2022; Hochhut et al., 2001). In this study, several antibiotics remained highly effective against the investigated isolates. All strains were susceptible to ciprofloxacin, trimethoprim–sulfamethoxazole, tigecycline, and amikacin, suggesting that these agents may still be effective treatment options for *V. cholerae* infections in this region. By contrast, high resistance rates were observed for colistin, streptomycin, chloramphenicol, and azithromycin, which is consistent with previous reports of *V. cholerae* from both clinical and environmental sources (Verma et al., 2019; Luo et al., 2021). Notably, environmental isolates exhibited higher resistance rates to several antibiotics, including chloramphenicol, streptomycin, and azithromycin, compared with clinical isolates. The consistently higher resistance rates in environmental isolates reinforces the role of aquatic environments as reservoirs of antimicrobial resistance in *V. cholerae* populations (Mageto et al., 2025; Li et al., 2025).

Genotypically, the distribution of antimicrobial resistance genes was generally consistent with the phenotypic resistance profiles. A high prevalence of quinolone resistance genes (*qnr*), tetracycline resistance genes (*tet*), and macrolide resistance genes (*mph*) was observed, particularly among multidrug-resistant isolates such as VC0861, VC0898, VC0899, VC0901, VC0902, and VC0904. However, a discrepancy was noted for β-lactam antibiotics: although reduced susceptibility was observed phenotypically, no β-lactamase genes (*bla*) were detected in the genomes. This phenotype–genotype inconsistency suggests that alternative resistance mechanisms, such as efflux pump overexpression (e.g., AcrAB-TolC) (Verma et al., 2019) or alterations in outer membrane porins (e.g., OmpU and OmpT) (Pauzé-Foixet et al., 2024), may contribute to the observed resistance.

The SXT element is a ~100-kb integrative and conjugative element that confers resistance to multiple antibiotics, including streptomycin, sulfamethoxazole, and trimethoprim (Beaber et al., 2002; Bioteau et al., 2018). The SXT element was identified in a clinical *V. cholerae* O139 strain in India and such elements have since been widely reported in both clinical and environmental isolates and are known to contribute to the dissemination of antimicrobial resistance among seventh-pandemic *V. cholerae* lineages (Hochhut and Waldor, 1999; He et al., 2021; Mohanraj and Mandal, 2022). Related elements have also been identified in other marine bacteria, such as *Photobacterium damselae* and *Shewanella putrefaciens* (Wozniak et al., 2009). In the present study, the SXT integrative element (*intSXT*) was detected in only one isolate (VC0827). This limited distribution suggests that horizontal gene transfer may contribute to the acquisition of resistance determinants in specific strains rather than being widespread across the population.

Virulence gene analysis identified a conserved set of virulence-associated genes (*als*, *hlyA*, *mshA*, *toxR*, and *rtxA*) that were present across all isolates in this study, consistent with their established roles in environmental persistence and host colonization (Montero et al., 2019). One clinical O139 isolate, VC0827, carried the *ctxAB* genes along with multiple virulence-associated genes, including *zot*, *ace*, *makA*, *ompU*, *toxR*, and *rtxA*. The presence of the VC2346 marker, which is associated with seventh-pandemic lineages, suggests that this strain may be related to pandemic-associated genotypes (Luo et al., 2024). In contrast, none of the isolates carried type III secretion system (T3SS) genes, while only a subset of strains possessed type VI secretion system (T6SS)-associated genes, indicating potential differences in virulence strategies and ecological adaptation (Luo et al., 2021; Suarez et al., 2008; Bhandari et al., 2023). In this study, the majority of O1 *V. cholerae* isolates carried partially or fully acquired pathogenicity islands; however, most lacked seventh pandemic islands. Only two strains, VC0298 and VC0827, harbored these elements. The acquisition of such pathogenicity islands may contribute to enhanced virulence potential and influence the distribution of pathogenic lineages (Montero et al., 2019). In this study, VC0827 carried a single copy of the *ctxB* gene, whereas multiple copies have been reported in other *V. cholerae* strains (Bhandari et al., 2023; Luo et al., 2024). Notably, VC0827 harbored three copies of ace and two copies of *zot*, in contrast to the single-copy arrangement observed in the reference strain E1162 (Hu et al., 2016). Such gene duplication may enhance toxigenic potential, although functional validation is required. Interestingly, VC0827 carried CTX prophage-associated genes (*ctxA*, *ctxB*, *zot*, and *ace*) in the absence of *tcpA*, which encodes the receptor required for CTX prophage integration. Similar observations have been reported in previous studies, suggesting that CTX-related elements can be acquired independently of TCP-mediated entry (Chakraborty et al., 2000; Menezes et al., 2014). One possible explanation is that CTX prophage integration may occur via host recombination systems, such as XerCD-mediated recombination at the chromosomal dif site (Das et al., 2014). Comparative analysis indicated that the CTX prophage region in VC0827 shares high similarity with those of previously reported epidemic strains, supporting its association with pandemic lineages (Waldor and Mekalanos, 1996; Rouard et al., 2022). Together, these findings highlight the potential role of phage-mediated gene transfer in the evolution and dissemination of toxigenic *V. cholerae*.

In addition to chromosomal resistance determinants and integrative elements, plasmid-mediated resistance plays an important role in the evolution of antimicrobial resistance. In this study, an IncC plasmid was identified in strain VC0827 and designated pVC0827. Previous reports have described multidrug-resistant plasmids in *Vibrio* species conferring resistance to antibiotics such as tetracycline, ampicillin, kanamycin, streptomycin, gentamicin, and trimethoprim (Luo et al., 2024; Luo et al., 2022). Consistent with this, pVC0827 carried genes conferring resistance to streptomycin, tetracycline, and sulfamethoxazole. Comparative analysis showed that pVC0827 shared substantial sequence similarity with the plasmid pVP205-NDM from *V. parahaemolyticus*, which carries a broad spectrum of resistance genes, including *bla*_NDM_, *tet*, and *emrE*. Although *bla*_NDM_ was absent in pVC0827, it contained multiple antimicrobial resistance genes, including *tet(A/B/M)*, *flor*, *sul2*, and *msr(E)*. IncC plasmids are broad-host-range conjugative plasmids widely distributed among Gram-negative bacteria and frequently associated with multidrug resistance (Carraro et al., 2016; Lassalle et al., 2023). Although conjugation-related genes (e.g., *tra* gene clusters) (Xu et al., 2024) were not specifically analyzed in this study, IncC plasmids are known to possess self-transmissible machinery that facilitates horizontal gene transfer (Fraga-Pampín et al., 2025). Therefore, such plasmids may mediate the interspecies dissemination of antimicrobial resistance determinants, contributing to the emergence of multidrug-resistant pathogens in both clinical and environmental settings. In this context, the presence of pVC0827 in a *ctxAB*-positive O139 strain indicates potential for the spread of resistance genes within *V. cholerae* populations and across aquatic microbial communities.

The cgMLST-based clustering analysis grouped the isolates into three main clusters, with Group 1 further subdivided into two distinct clinical clusters. VC0827 grouped closely with reference seventh-pandemic strains, such as E1162, and shared the same sequence type (ST69), supporting its genetic relatedness to pandemic lineages (Luo et al., 2024; Hu et al., 2016). By contrast, other isolates formed separate clusters characterized by lower virulence gene content and more diverse resistance profiles, particularly among environmental strains in Group 3. This genetic differentiation suggests ongoing diversification of *V. cholerae* in both clinical and environmental settings, potentially driven by lineage expansion and horizontal gene acquisition.

## 5 Conclusion

In summary, this study provides a comprehensive characterization of *V. cholerae* O1 and O139 isolates collected in Jiaxing, China, between 2021 and 2024 using antimicrobial susceptibility testing and whole-genome sequencing. The isolates exhibited diverse antimicrobial resistance patterns and virulence gene profiles, with several environmental strains showing higher resistance rates than clinical isolates. Notably, one clinical O139 strain (VC0827) was identified as *ctxAB*-positive and carried an IncC plasmid harboring multiple antimicrobial resistance genes. Genomic analysis suggested that VC0827 is related to seventh-pandemic *V. cholerae* lineages. Overall, these findings demonstrate the genomic diversity of *V. cholerae* circulating in both clinical and environmental settings in Jiaxing and underscore the importance of continuous genomic surveillance to monitor the emergence of toxigenic and multidrug-resistant strains, as well as the potential role of horizontal gene transfer in shaping the evolution of pathogenic *V. cholerae*.

## Data Availability

All raw data have been uploaded to NCBI under accession number PRJNA1437248.

## Footnotes

1
https://cge.food.dtu.dk/services/ResFinder/

2
http://bacdb.cn/BacWGSTdb

3
https://itol.embl.de

4
https://phaster.ca/

5
https://cge.food.dtu.dk/services/PlasmidFinder/

6
https://www.ncbi.nlm.nih.gov/nucleotide/
